# The evaluation of an online mindfulness program for people with multiple sclerosis: study protocol

**DOI:** 10.1186/s12883-019-1356-9

**Published:** 2019-06-14

**Authors:** Amy-Lee Sesel, Louise Sharpe, Heidi N. Beadnall, Michael H. Barnett, Marianna Szabo, Sharon L. Naismith

**Affiliations:** 1 School of Psychology, University of Sydney, Sydney, NSW 2006 Australia; 2 Brain and Mind Centre, University of Sydney, Sydney, NSW 2006 Australia; 3 Neurology Department, Royal Prince Alfred Hospital, Camperdown, NSW 2050 Australia

**Keywords:** Multiple sclerosis (MS), Randomized controlled trial (RCT), Mindfulness, Depression, Anxiety, Fatigue, Pain, Health-related quality of life

## Abstract

**Background:**

Multiple sclerosis (MS) is a neurological disease of the central nervous system and is associated with many psychosocial symptoms that are difficult to manage including low mood, anxiety, fatigue and pain, as well as low health-related quality of life. Internet-based psychosocial interventions that use mindfulness-based approaches are gathering much attention in recent literature, particularly in the treatment of chronic illnesses. However, no large randomized controlled trials have been done examining the efficacy of such interventions for people with MS (PwMS).

**Methods/design:**

This study is a randomised controlled trial of an online mindfulness-based intervention (MBI) for PwMS. Participants will be randomised to receive either the MBI or offered the intervention after a waiting period. All participants will be assessed to determine whether they have a history of recurrent depressive disorder. The primary outcome will be severity of depression, according to the Centre of Epidemiology Depression Scale. Secondary outcomes will be anxiety severity, fatigue, pain and quality of life. Assessments will be conducted pre, post-treatment, at three and six-month follow-up. The online mindfulness-based program was developed in collaboration with end-users (*n* = 19 PwMS) who gave feedback about what would be feasible and acceptable, and the draft program was reviewed by both experts and patients.

**Discussion:**

Multiple sclerosis is the most common acquired chronic neurological disease amongst young adults and is associated with a range of symptoms that can be difficult to cope with. In face-to-face interventions, a MBI demonstrated the largest effect in a recent meta-analysis of psychological treatments for PwMS, but MBIs for PwMS have not been delivered online. Hence, this trial will confirm whether MBIs can be efficacious when delivered online. A range of symptoms are assessed as outcomes so that the nature of benefits associated with the online MBI can be ascertained.

**Trial registration:**

ACTRN12618001260213.

**Date of Registration:** 25/07/2018.

**Electronic supplementary material:**

The online version of this article (10.1186/s12883-019-1356-9) contains supplementary material, which is available to authorized users.

## Background

Multiple sclerosis (MS) is an autoimmune demyelinating disease of the central nervous system [1]. While it remains a clinical diagnosis, the diagnostic criteria for MS has evolved over the decades and recent iterations have been more influenced by radiological findings, namely the identification of lesions or plaques in the brain and/or spinal cord in a particular distribution and with certain features [2]. The 2017 McDonald Criteria for MS are the most recent diagnostic criteria, and radiological features have been incorporated to allow an earlier diagnosis of MS than with earlier versions of the diagnostic criteria [2]. People with MS (PwMS) have a range of psychosocial consequences and symptoms associated with the illness that compromise their quality of life. These include depression, anxiety, fatigue and pain. The most commonly used psychosocial approach to improve coping with these symptoms is Cognitive Behaviour Therapy (CBT). Early meta-analyses found that CBT is effective for improving mood [3–5], fatigue [6], and health-related quality of life (HRQoL) [7] in PwMS. However, these meta-analyses included relatively few studies, most with small sample sizes (e.g. *n* < 20 per study arm) which may over-estimate treatment efficacy [8]. Some only included studies that pre-screened for low mood [3, 4]. A more recent, comprehensive meta-analysis examined the efficacy of psychosocial interventions for PwMS [9] and found that CBT was not effective in improving anxiety, depression, fatigue or HRQoL in PwMS, when considered in isolation, in contrast to other interventions.

From the most recent meta-analysis, the intervention with the largest effect sizes for depression (*d* = 0.8) and anxiety (*d* = 0.6) in PwMS was an 8-week mindfulness-based approach [10], which was based on Jon Kabat-Zinn’s mindfulness-based stress reduction (MBSR) program [11]. This intervention also produced moderate effects for fatigue (*d* = 0.5) and HRQoL (*d* = 0.6) in PwMS, suggesting that a mindfulness program may be an appropriate psychosocial intervention to investigate as an adjunctive treatment to the medical management of MS.

Studies have shown that mindfulness-based interventions, such as MBSR, are efficacious for a wide spectrum of chronic disorders including fibromyalgia [12], rheumatoid arthritis [13], coronary artery disease [14], cancer [15], chronic pain [16], chronic fatigue [17] depression and anxiety [18]. However, Grossman and colleagues’ [10] study is the only published randomized controlled trial that examined the effect of mindfulness-based interventions for PwMS on a range of health outcomes including depression, anxiety, fatigue, pain and quality of life. This high quality randomized controlled trial had a relatively large sample of 150 PwMS. It was delivered face-to-face and it was intensive (27 hrs in total). For many PwMS, such a lengthy program would be difficult to access for a variety of reasons including work status, rurality, financial barriers and/or disability. Indeed, due to the chronic and potentially disabling nature of MS, frequent or regular travel can be difficult. Numerous previous studies have relied upon psychological interventions being provided over the telephone [19–21] or via teleconference [22]. Furthermore, a 2015 survey of over 2800 PwMS found that there was a considerable unmet psychological need to optimise the accessibility of psychological services and support [23].

One mode of treatment delivery that may be easily transferable to clinical practice, but which is yet to be fully explored for PwMS, is Internet-delivered therapy. There is considerable evidence that online interventions guided by a therapist result in improvements that are equivalent to face-to-face formats for a range of mental and physical health problems [24]. So far, there are three trials investigating the efficacy of online psychological interventions for PwMS. All three have used a cognitive-behavioural approach. The first was a proof of concept trial (*n* = 24), called “Beating the Blues” [25] which reported a number of difficulties including a low recruitment rate and no outcome data were reported. The "MS Invigor8" trial, [26] was also a pilot (*n* = 40), but reported preliminary outcome data and found improvements in fatigue, anxiety, depression, quality of life, as well as good acceptability of the online program. The "Deprexis" trial, by Fischer et al., (2015) was a larger RCT (*n* = 90) [27] that evaluated a standard iCBT protocol for depression in PwMS. They found significant improvements in depressive symptoms but not in the disease-specific quality of life measure, and only the psychological well-being subscale of a general quality of life scale. Fischer and colleagues reported that participants indicated the need to tailor the program to MS-specific needs. The authors also speculated that their treatment effects may have been enhanced by the inclusion of therapist support. Taken together, these online CBT studies indicate that Internet-delivered psychosocial interventions for PwMS may be an acceptable and viable option for PwMS.

Whilst CBT is considered to be the “gold standard” psychological therapy for a range of psychological indications associated with MS, our meta-analysis [9] identified that CBT may not be an optimal approach for PwMS. CBT is typically a short-term intervention, and relies on the ability to think analytically, focusing on identifying and challenging unhelpful beliefs. These skills may be suboptimal for those with cognitive impairment, which is often apparent in PwMS (ranging from 43 to 70%, depending on the setting [28]). In contrast, mindfulness-based interventions involve the long-term practice of meditation, but focus on the development of a single skill (i.e. cultivating a moment-to-moment awareness of the present moment), which may be more appropriate for people with MS and concomitant cognitive decline. Furthermore, these “mind-body” interventions, involving “body-based”, experiential learning promote acceptance and the development of an increased tolerance (or desensitization) to difficult thoughts, emotions and sensations, which may be more suited to PwMS who are faced with the unrelenting challenges of adjusting to life with an irreversible and progressive chronic illness.

### Aims and hypotheses

The aim of the current study is to evaluate the efficacy of an online mindfulness-based program for symptoms of depression, anxiety, fatigue, pain and health-related quality of life in PwMS. Whilst mindfulness is a centuries-old tradition stemming from Buddhist philosophy, the current scientific interest in mindfulness as a treatment for mental health problems started as a method to prevent depressive episodes in people with a history of depression who were currently asymptomatic. Evidence supports the efficacy of mindfulness for the prevention of recurrence of depression [29]. Since a large proportion of PwMS develop major depression (50% lifetime prevalence [30]), the ability of mindfulness to prevent future relapse of depression amongst those with a history of depression may be particularly valuable in the management of PwMS. Furthermore, whilst Grossman et al. (2010) did not examine depressive disorders or relapses of depression, there is evidence in the rheumatoid arthritis (RA) literature that while CBT was more effective overall than MBSR, for those patients who had a history of recurrent depression (two or more episodes), mindfulness outperformed CBT [31]. Given that the prevalence of clinically significant depression in people with rheumatoid arthritis is estimated to be approximately 16% [32] and the rate is considerably larger for PwMS, the potential benefits of mindfulness may be greater in MS. Therefore, a secondary aim of this study is to determine whether PwMS with a history of recurrent depression will benefit significantly more from the online mindfulness program than those without. We define recurrent depression as having two or more episodes of depression, consistent with work in RA [31]. If there are insufficient numbers to test the recurrent depression hypothesis, we will examine whether having any history of lifetime depression (current or history) is a moderator of treatment effect. To elucidate the likely mechanisms of treatment gains, we will examine whether changes in the state mindfulness will mediate the effect between treatment group and treatment outcomes, and whether adherence to mindfulness practice predicts outcome in the treatment group.

We hypothesize that **(1)** PwMS who participate in the online mindfulness intervention will demonstrate significant improvements in depressive symptoms (primary outcome), anxiety, fatigue, pain and HRQOL outcomes at post-treatment, 3-month, and 6-month follow-up, compared with waitlist controls; **(2)** PwMS with a history of recurrent depression will benefit significantly more from the online mindfulness program than those without; **(3)** Changes in mindfulness will mediate the effect between treatment group and treatment outcomes; **(4)** PwMS that report greater meditation adherence will have greater improvements in outcomes than those without.

## Methods/design

**Fig. 1:**
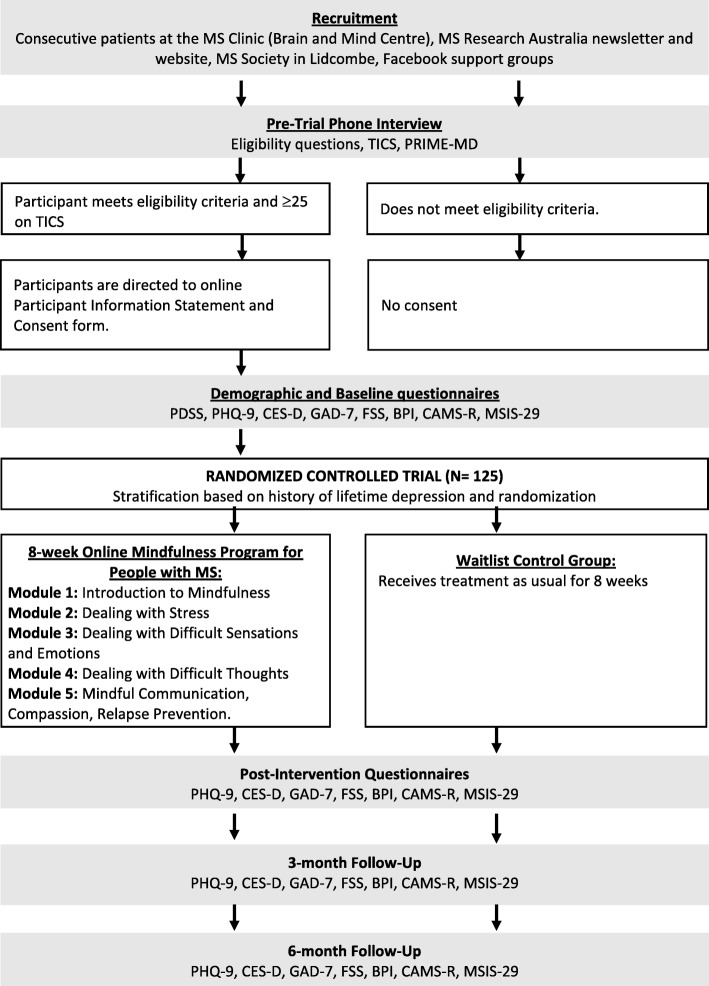
Outline of study design. TICS; Telephone Interview for Cognitive Status, PRIME-MD; Primary Care Evaluation of Mental Disorders- Major Depression Module, PDDS; Patient Determined Disease Steps, PHQ-9; Patient Health Questionnaire, CES-D; Centre for Epidemiological Studies of Depression, GAD-7; Generalized Anxiety Disorder 7-item scale, FSS; Fatigue Severity Scale, BPI-SF; pain intensity and interference from the Brief Pain Inventory- Short Form, MSIS-29; Multiple Sclerosis Impact Scale, CAMS-R; Cognitive and Affective Mindfulness Scale- Revised

The current study will be a CONSORT-R compliant [33] randomized controlled trial (RCT) and is registered with the Australian New Zealand Clinical Registry (ACTRN12618001260213). Ethics approval has been granted by the University of Sydney Human Research Ethics Committee (Project No. 2018/402). Please see Fig. 1 for an illustration of the trial procedure.

### Participants

People with MS will be included in this study if they (1) report a neurologist-confirmed diagnosis of MS (2) ≥ 18 years old (3) currently live in Australia (4) have regular access to the Internet (5) have sufficient English to complete questionnaires and understand program content in English (6) if taking MS treatment, are on a consistent MS regimen for > 1 month (7) if taking anti-depressant medication, are on a stable dose for > 8 weeks. Participants will be excluded if they have (1) a comorbid medical condition known to impact on cognition or physical ability (2) moderate-severe cognitive deficits (< 25 on the Telephone Interview for Cognitive Status; TICS [34]) (3) suicidal intent requiring emergency care (4) alcohol or drug abuse or dependence (5) a psychotic illness (6) received consistent psychotherapy within the last 6 months (7) are pregnant.

Participants will be recruited from the Multiple Sclerosis Clinic at the Brain and Mind Centre, the University of Sydney (Sydney, Australia), by (1) contacting patients that expressed interest in being involved in “future mindfulness-based studies” as part of a previous qualitative study; and (2) approaching consecutive patients face-to-face. In addition, the study will be advertised by Multiple Sclerosis Research Australia via newsletters and clinical trial websites, through flyers posted at the MS Society, as well as in Multiple Sclerosis Facebook support groups.

### Procedure

Participants that have registered interest in the study will be contacted using contact details provided on an online form. A pre-trial telephone interview will be conducted by a registered psychologist (supervised by clinical psychologist, LS) to establish eligibility for the study. Participants that meet criteria will undergo a telephone-administered clinical interview, the Primary Care Evaluation of Mental Disorders- the Major Depression Module (PRIME MD; [35]) to determine those with a history of lifetime depression and those without. PwMS that indicate that they have suicidal intent and require emergency care will be referred to the appropriate mental health service according to a suicide risk assessment decision tree and will not be eligible to participate.

Participants will be given information about the study over the phone and online. They will also be able to provide consent to the study online, and will be sent the Participant Information and Consent forms (see Additional file 1) via e-mail for personal record-keeping. Those that consent will be automatically directed to demographic and baseline questionnaires (Patient Health Questionnaire; PHQ-9 [36]; Centre for Epidemiological Studies of Depression; CES-D [37]; Generalized Anxiety Disorder Scale; GAD-7 [38]; Fatigue Severity Scale; FSS [38]; Pain intensity and interference from the Brief Pain Inventory- Short Form; BPI-SF [39]; Multiple Sclerosis Impact Scale; MSIS-29 [40]; 10-item Cognitive and Affective Mindfulness Scale- Revised; CAMS-R [41]). Upon completion, randomisation will be conducted by an independent researcher into either the 8-week mindfulness-based intervention or a waitlist control group. Participants will then be contacted by a member of the research team and informed of their randomised condition.

Participants allocated to the mindfulness-based intervention will receive five modules (approx. 15 mins in length), followed by a multiple-choice module quiz (approx. 1 min), over 8 weeks (1 module per 1–2 weeks) and five accompanying meditation audio-guides. They will also be guided by 5–8 × 10-min telephone calls (approx. 1 per week) with a registered psychologist to (1) encourage participation (2) normalize challenges to treatment (3) help resolve any technological difficulties. Participants allocated to the waitlist group will not receive the intervention, but will continue having treatment as usual, and will be asked to complete all demographic and outcome questionnaires. All participants will complete the Patient Health Questionnaire (PHQ-9 [36]) at baseline and every week online, throughout the course of the trial, as well as at post-intervention, 3-month and 6-month follow- up) to monitor mood and suicide risk. Post-intervention questionnaires will be administered to all participants at post-intervention (Week 9), at 3-month follow-up (Week 21) and at 6-month-follow-up (Week 33). Those who do not complete will be contacted by email and/or telephone up to three times in order to remind them complete their questionnaires.

### Randomisation

Randomisation will be stratified by history of major depression (assessed via the PRIME-MD). This will be performed by a researcher who will have no contact with participants throughout the duration of the trial, using computer-generated random numbers.

### Therapist training and trial conditions

The online mindfulness program will be guided by a registered psychologist (AS). These telephone calls will be used to provide support and encouragement and ensure understanding, but will not be used to provide therapy. The project will follow strict manualised protocols under the supervision of a clinical psychologist (LS. Any adverse events will be promptly reported using the Adverse Events reporting guidelines, to the University of Sydney, Human Research Ethics Committee.

### Mindfulness intervention

The online program has been manualized and adapted from Dr. Jon Kabat Zinn’s MBSR program [11]. It was developed by a registered psychologist (AS) with training in both clinical psychology and mindfulness. It was tailored to the needs and attitudes of PwMS based on a series of 19 face-to-face qualitative interviews that were thematically analysed according to their psychological experiences, difficulties with MS-related symptoms, and responses to questions about the acceptability of the proposed online mindfulness program. The resultant online modules contain fictional case examples of unique characters with different symptom clusters that vary in terms of gender, race, age, disease course and duration that were derived from the interviews. These case examples demonstrate how MBSR techniques can be used to manage a variety of problems that many PwMS face. The five main topics areas of the modules broadly cover: 1) An Introduction to Mindfulness Meditation, 2) Dealing with Stress, 3) Dealing with Difficult Sensations and Emotions, 4) Dealing with Difficult Thoughts, and, 5) Mindful Communication, Compassion and Relapse Prevention. Once created, these modules were then modified and revised based on the suggestions, comments and feedback from a subsample of the original 19 PwMS who viewed the program (*n* = 11). The program was also reviewed by a range of experts (*n* = 8) including three clinical psychologists, two of which have knowledge and extensive training in mindfulness meditation, one clinical neuropsychologist, two neurologists who specialize in MS, one academic emergency physician who has knowledge and experience in the medical treatment of MS and in teaching mindfulness meditation to PwMS, as well as one academic general practitioner who is a mindfulness expert with extensive experience in coordinating the online delivery of mindfulness-based interventions. The three lead authors (AS, LS and SN) then met to discuss each feedback point and determine whether to amend the program in response to that specific feedback.

The mindfulness meditation audio-guides were based on Dr. Jon Kabat Zinn’s meditation scripts (e.g. Awareness of Breath, Body Scan meditation) and were slightly adapted in the introduction to remind participants about the corresponding module. Suggestions for informal mindfulness practice were also included at the end of each module e.g. “Choose an activity this week that you can do mindfully”, and Mindfulness Logs were created as part of the program to help participants record the type, frequency and duration of meditation practice each week.

### Outcome measures

The primary outcome measure will be depressive symptoms, as measured by the CES-D [37]. The secondary outcomes will include depressive symptoms (PHQ-9), and will be measured online, at baseline, Weeks 1- Week 8, at post-intervention, 3-month and 6-month follow up. All other secondary outcomes, including anxiety (GAD-7 [38]); fatigue (FSS [38]); pain (BPI-SF; [39]), and MS-related Quality of Life (MSIS-29 [40]) and the primary outcome will be measured online at baseline, post-intervention, 3-month and 6-month follow-up.

To investigate factors that may affect treatment success, we will also be measuring several process outcomes. A measure of mindfulness (CAMS-R 10-item [41]) will be administered to all participants at baseline, post- intervention, 3-month and 6-month follow-up in order to investigate whether changes in mindfulness will mediate the relationship between treatment group and treatment outcomes. At post-intervention (Week 9), participants will also be asked to answer online questions about any changes to treatment during the past eight weeks, including whether they started or changed any disease-modifying MS treatments, started seeing a psychologist/ psychiatrist, began or changed the dose of any new mood stabilizing treatments. Participants in the intervention group will complete additional therapy adherence questions (i.e. the type, frequency, and duration of mindfulness meditation) at the end of each week, at 3 and 6-month follow-up. Those in the intervention group will also complete the Credibility/Expectancy questionnaire [42] at the end of Week 1 and questions regarding treatment satisfaction during the last week of the program (Week 8).

The demographic questionnaire administered at the outset of the trial will include questions about patient characteristics (e.g. age, gender, employment status), disease characteristics (e.g. type, duration of MS), current MS-related and mood-stabilizing treatments, and previous experience with psychological therapy and mindfulness meditation. Confirmation of MS diagnosis and type of disease course will be obtained with participants’ permission from their neurologist. The Patient Determined Disease Steps (PDDS) questionnaire [43] will also be administered as part of the demographic data, as a measure of self-reported disability in order to determine whether level of disability moderates treatment outcome.

## Analysis

### Sample size

Our sample size calculation is based on Sesel and colleagues’ [9] meta-analysis that found effect sizes of psychosocial interventions ranging from (*d* = 0.2–0.3). According to G*Power, we would need a total sample of 125 to get 80% power with an alpha of .05 including an expected 20% drop-out rate.

### Statistical analysis

Data will be analysed using intention-to-treat analysis [44]. There are four measurement times, of which three are post-intervention. Linear mixed models will be generated with group as the fixed factor and baseline level of each treatment outcome (e.g. CES-D, GAD-7) as the covariate. Multiple linear regression analyses will also be used to predict change in outcomes over treatment and to investigate the predictive value of potential moderators (i.e. type of MS, depression, fatigue and pain status). Missing data will be minimized using a forced online completion format and participants who are willing will be asked to complete questionnaires even if they drop-out from the online program. Reporting will follow the CONSORT statement [33]. For further information on data management and security, please see Additional file 1.

## Discussion

MS is a potentially debilitating disease and can be associated with increasing disability in the 10–20 years post diagnosis, especially if disease modifying therapy is not commenced early, is ineffective or is not used at all [45, 46]. MS is typically diagnosed between the ages of 20 and 40, with a huge majority falling within the working age (87%) [47]. In Australia, as with the rest of the world, the financial burden of having MS is high, with Australians with MS spending in total $78 million out of their personal funds to seek treatment and pay for other health care expenses [46, 47]. Recent research suggests large and broad benefits from mindfulness-based interventions, but these are rarely available to people with MS. Barriers include difficulties with mobility due to the illness and associated disability. Other barriers likely include cost, time and geographical location [48]. Internet-based psychological interventions may not only help to alleviate the psychological and economic burden of being faced with increasing disability and loss of employment, but may offer working adults the flexibility of being able to access therapy outside of working hours or at times that are best suited to their needs and responsibilities.

The proposed study will be the first to evaluate an online mindfulness-based program that is tailored for PwMS and targets a range of psychosocial symptoms and consequences associated with the disease, including depression, anxiety, fatigue, pain and health-related quality of life. The program is likely to be cost-effective, rendering it more accessible than traditional face-to-face interventions. Further, online interventions are manualized with minimal therapist input, rendering them reproducible and more amenable to future research. The eventual goal of this study is to make available an online, evidence-based psychological intervention for PwMS that is scalable and thus could be distributed through service provision to improve symptoms of depression, anxiety, fatigue, pain and quality of life.

## Conclusion

People with MS may experience a wide range of physical and psychosocial symptoms that are difficult to manage. Given the unpredictable nature of the disease, psychological interventions targeted towards reducing symptoms of depression, anxiety, fatigue and pain may help to alleviate emotional distress associated with the possibility of increasing disability and optimise quality of life. Whilst CBT is widely regarded as the “gold standard” psychological therapy, our recent meta-analysis [9] indicated that this type of therapy may not be beneficial for people with MS. Thus, further investigation into alternative types of therapy is needed. In recent years, there has been good evidence for the efficacy of mindfulness-based therapies for people with chronic health conditions. Furthermore, there is considerable evidence that guided interventions delivered online result in improvements that are equivalent to face-to-face formats for a range of mental and physical health problems. Online therapies are more likely to be cost-effective, and allow greater access to treatment for people that live remotely, as well as for those whose mobility is affected by illness, such as many PwMS. Research into the efficacy of an online mindfulness-based program for PwMS may be transferred into clinical practice and serve as an important adjunct to the medical management of the disease.

## Additional file

Additional file 1: Participant Information Statement and Consent Form (DOC 379 kb)

## Data Availability

Data from this research will be made available for research purposes. Requests (incl. a synopsis of the planned research) can be addressed to the corresponding author.

## Abbreviations

BPI-SF: Brief Pain Inventory- Short Form
CAMS-R: Cognitive and Affective Mindfulness Scale- Revised
CBT: Cognitive Behavioural Therapy
CES-D: Centre for Epidemiological Studies of Depression
FSS: Fatigue Severity Scale
GAD-7: Generalized Anxiety Disorder 7-item Scale
HRQOL: Health-related quality of life
MBI: Mindfulness-based intervention
MBSR: Mindfulness-Based Stress Reduction
MS: Multiple sclerosis
MSIS-29: Multiple Sclerosis Impact Scale
PRIME-MD: Primary Care Evaluation of Mental Disorders
PwMS: People with multiple sclerosis
RCT: Randomised Controlled Trial
TICS: Telephone Interview for Cognitive Status
