# Supplementary material for: The evaluation of an online mindfulness program for people with multiple sclerosis: study protocol
Source: BMC Neurol. 2019 Jun 14;19:129. doi: 10.1186/s12883-019-1356-9 (PMC6567500; doi:10.1186/s12883-019-1356-9)
Supplement: Supplementary file 1 — Participant Information Statement and Consent Form (DOC 379 kb) [file 12883_2019_1356_MOESM1_ESM.doc]

Appendix I: Participant Information Statement and Consent Form

| 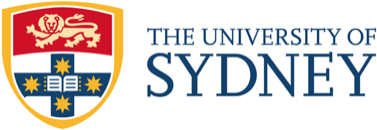 | |  | **School of Psychology Faculty of Science** |
| --- | --- | --- | --- |
|  | ABN 15 211 513 464 | |  |
|  | **PROFESSOR LOUISE SHARPE**  *Chief Investigator (Supervisor)* | | Room 450  Brennan MacCallum (A18)  The University of Sydney  NSW 2006 AUSTRALIA  Telephone: +61 2 9351 4558  Email: louise.sharpe@sydney.edu.au  Web: <http://www.sydney.edu.au/> |

**Online Mindfulness Program for People with MS**

**PARTICIPANT INFORMATION STATEMENT**

1. **What is this study about?**

You are invited to take part in a research study about ***mindfulness for people with Multiple Sclerosis. Mindfulness involves the daily practice of meditation, which is a mental exercise that is thought to improve the mind’s ability to focus, by paying attention to the present moment, without judgment. Our primary aim is to see whether an online 8-week mindfulness-based program improves outcomes for people with MS.***

You have been invited to participate in this study because ***you have expressed interest in this study and meet eligibility criteria including but not limited to: having a neurologist-confirmed diagnosis of Multiple Sclerosis, living in Australia, being ≥ 18 years old and having regular access to the Internet.*** This Participant Information Statement tells you about the research study. Knowing what is involved will help you decide if you want to take part in the research. Please read this sheet carefully and ask questions about anything that you don’t understand or want to know more about.

Participation in this research study is voluntary.

By giving your consent to take part in this study you are telling us that you:

- Understand what you have read.
- Agree to take part in the research study as outlined below.
- Agree to the use of your personal information as described.

You will be given a copy of this Participant Information Statement to keep.

1. **Who is running the study?**

The study is being carried out by the following researchers:

- Professor Louise Sharpe (School of Psychology, The University of Sydney)
- Professor Sharon Naismith (School of Psychology, The University of Sydney).
- Dr Michael Barnett (Medicine, Central Clinical School, The University of Sydney)
- Dr Heidi Beadnall (Medicine, Central Clinical School, The University of Sydney)
- Amy-Lee Sesel (School of Psychology, The University of Sydney)

Amy-Lee Sesel is conducting this study as the basis for the degree of her PhD degree at The University of Sydney. This will take place under the supervision of Professor Louise Sharpe, Professor of Clinical Psychology, School of Psychology, The University of Sydney. The project is being supported by a co-funded postgraduate scholarship awarded to Amy-Lee Sesel from the National Health and Medical Research council and Multiple Sclerosis Research Australia.

1. **What will the study involve for me?**

We are conducting a randomised controlled trial to investigate the efficacy of an 8-week online mindfulness program for people with MS. This means that if you decide to take part, there is a 50% chance that you will be asked to complete the 8-week online program immediately and a 50% chance that you will be in the waitlist group. Neither you nor the researchers will be able to choose whether you are allocated to get the program immediately or after a period of waiting.

- *If you are randomly allocated the 8-week online program:* You will be sent 5 online modules (approx. 15mins each) over an 8-week period detailing your mindfulness meditation practice for each week. You will have access to 30-min audio-guides that you will be encouraged to listen to daily as well as Mindfulness Logs, which you may use to schedule and track your mindfulness progress. You will also receive 5-8 phone calls (5-10mins each) throughout the program (approx. 1 per week) from a psychologist who is part of the research team to help you resolve any difficulties you have and to answer any of your questions.
- *If you are randomly allocated the waitlist group and the treatment is found to be effective*: You will be offered the online program after 6 months, where you can complete it on your own, at your own pace.

No matter which group you are assigned, ALL participants will be asked to complete a brief battery of online questionnaires asking you about symptoms of depression, anxiety, fatigue, pain, quality of life and mindfulness at:

- Baseline,
- Week 9,
- Week 21 (3-month follow-up), and
- Week 33 (6-month follow-up).

Links to all the online questionnaires will be sent to you via e-mail. We will make a friendly 5-min phone call to you to remind you to complete the questionnaires if they have not done so.

Throughout the program, all participants will be asked to complete short (approx. 5 min) online questionnaire each week to monitor your mood. During the final week, additional questions (approx. 5mins) will be asked about your relationship with the psychologist running the program, and any changes to treatment. If you are in the 8-week online program group will also be asked questions about your mindfulness practice and module content each week, as well as treatment satisfaction questions at the end of the program, which will only take an additional 1-3 mins more or your time.

If you decide to participate in this research after reading this online form and clicking “I consent”, you will be directed to a set of online questionnaires including:

- Demographic questions (e.g. age, marital status, education and disability status),
- Questions about your disease characteristics (e.g. type of MS, time since diagnosis, number of relapses),
- Current MS-related and/or mood stabilizing medication (if applicable),
- Previous experience with psychological therapy, the Internet, and mindfulness meditation, followed by
- Baseline questionnaires (as described above).

Once you have complete these questionnaires, we will send you an e-mail, welcoming you to the study, informing you of which group you have been randomly allocated and the study program.

Please see below for a diagram of the Study Flowchart.

You will be randomized to either the 8-week online program or waitlist and will be sent an e-mail, informing you of which group you have been allocated, and the study program.

Randomization into two groups and stratification based on history of depression as indicated on the PRIME-MD

If you consent to participate in this study, you will be directed to:

**Demographic and Baseline questionnaires:**

Questions about symptoms of depression, anxiety, fatigue, pain, quality of life, mindfulness quality of life.

**Mindfulness Program:**

5 online modules,

30-min daily meditation + weekly brief questionnaires / 8 weeks

**Waitlist group**

Weekly brief questionnaires to monitor mood / 8 weeks

**Week 9 Follow-up**

**3-Month Follow-up**

**6-Month Follow-up**

Approx. 30 mins

Approx. 5mins/ week

Approx. 30 mins

Approx. 30 mins

Approx. 30 mins

1. **How much of my time will the study take?**

It is estimated that each block of questionnaires (Demographic and baseline, Week 9 follow-up, 3-month follow-up and 6-month follow up), will take approximately 30mins to complete. During the first 8 weeks of the study, participants in the online Mindfulness program and in the waitlist group will be asked to take an additional 5mins each week (8 mins during the last week) to complete the weekly brief questionnaires. Thus,

- For participants in the 8-week online program, the study will involve 5x 15min online modules (1 hr, 15 mins) + 30min mindfulness meditation audio-guides daily x 8 weeks (28 hours) + 5-8min brief questionnaires x 8 weeks (48 mins) + 5-8 brief telephone calls (5mins each x 8 weeks) + 30min questionnaire blocks at baseline, Week 9, 3-month and 6-month follow-up (2 hours), totalling: approx. 33 hours over 6-months.
- For participants in the waitlist group, the study will involve: 5-8min brief questionnaires x 8 weeks (48 mins) + 30min questionnaire blocks at baseline, Week 9, 3-month and 6-month follow-up (2 hours), totalling: approx. 2 hours and 48mins over 6 months.

1. **Who can take part in the study?**

People with Multiple Sclerosis who live in Australia and are ≥ 18 years old are the population of interest for this study. People that are eligible to take part in this study need to:

1. Have a neurologist-confirmed diagnosis of MS
2. Be ≥ 18 years old
3. Currently live in Australia
4. Have regular access to the Internet
5. Have sufficient English language skills to complete questionnaires and understand the program in English
6. If taking MS treatment, be on a consistent MS regimen for more than 1 month with no plans to change this
7. If taking anti-depressant medication, a stable dose for > 8 weeks.

People that are not eligible for this study are those that:

1. Do not consent to the researchers contacting their neurologist to confirm their MS diagnosis.
2. Have another medical condition known to impact on cognition or physical ability (because we want to evaluate the efficacy of a program specifically designed for symptom management in MS only at this point)
3. Have moderate or severe cognitive problems (because we want to make sure that people will be able to fully understand and carry out the program in the way that is intended)
4. Have suicidal intent requiring emergency care (for safety reasons)
5. Have alcohol or drug abuse or dependence
6. Have a psychotic illness (for safety reasons)
7. Have received consistent psychotherapy within the last 6 months (because we want to know whether any psychosocial benefits identified are a result of the program itself and not previous psychological therapy).
8. Are pregnant (because pregnancy often improves MS and therefore could make it hard to tell if the program was effective or MS improved due to pregnancy). Please note that whilst, if you fall pregnant during the trial, this does not preclude you from participating.
9. **Do I have to be in the study? Can I withdraw from the study once I've started?**

Being in this study is completely voluntary and you do not have to take part. Your decision whether to participate will not affect your current or future relationship with the researchers or anyone else at the University of Sydney, or the MS Clinic (Brain and Mind Centre).

If you decide to take part in the study and then change your mind later, you are free to withdraw at any time. You can do this by e-mailing the researcher: [amy-lee.sesel@sydney.edu.au](mailto:amy-lee.sesel@sydney.edu.au) or calling: +61417 061 710

If you decide to withdraw from the study, your questionnaire responses cannot be withdrawn once they are submitted as they will become part of a pool of data and will be analysed accordingly.

In the rare case in which we feel we need to terminate your participation without your consent (e.g. serious adverse event or safety concerns), we will call you using the contact details provided and devise a safety management plan with you which may include a referral to an appropriate healthcare service to ensure your well-being and safety**.**

1. **Are there any risks or costs associated with being in the study?**

Although it is theoretically possible that the online mindfulness program could cause you distress, in this instance, prolonged distress is extremely unlikely. The online mindfulness program is adapted from Dr John Kabat-Zinn’s Mindfulness-Based Stress Reduction program, which is an evidence-based treatment for the management of stress, and other psychological outcomes. This program has been shown to reduce depressive symptoms, anxiety, and chronic pain in a number of clinical trials in people with chronic illnesses, including multiple sclerosis. The program will be administered by a registered psychologist under the supervision of an experienced clinical psychologist, both of whom are trained to manage distress and are also able to assess any risk to you. Whether you allocated the 8-week online program or waitlist group, your mood will be monitored throughout the duration of the study using a weekly online questionnaire so that we can identify if you are becoming more distressed over the course of the trial. If you indicate that you are experiencing severe symptoms of depression on this questionnaire or during your weekly phone calls with the psychologist (for those in the 8-week program), you will be contacted for further assessment, and referred to your GP or other appropriate services as needed.

There will be no financial costs involved in participating in this study. We anticipate that the most likely costs involved in participating in this study will involve giving up your time to participate in the program (e.g. practice mindfulness meditation daily) and/or completing the online questionnaires.

1. **What happens if I suffer injury or complications as a result of the study?**

If you suffer any injuries or complications as a result of this study, you should contact Amy-Lee Sesel via email: [amy-lee.sesel@sydney.edu.au](mailto:amy-lee.sesel@sydney.edu.au) or phone: +61417061710 and she will put you in contact with a doctor who will assist you in arranging appropriate medical treatment.

1. **Are there any benefits associated with being in the study?**

Whilst we cannot guarantee any direct benefits of participating in this study, potential benefits include having your mood monitored over the period of the study duration.

We hope that potential benefits of the study will include improvements in symptom management for people with MS if the program is found to be effective, in which case the results of this study will be used to further our understanding of mindfulness based interventions for people with MS.

1. **Will you be requiring any information from my neurologist (if available) in order to participate in this study?**

We would like to ask for your consent to contact your neurologist to a) confirm your diagnosis of MS and b) type of disease course c) date of diagnosis d) most current Expanded Disability Status Scale score. The confidentiality and privacy of your data will be upheld along with all the information that you provide as part of this research if you consent to the release of this information

1. **What will happen to information about me that is collected during the study?**

During this study, we will be collecting your demographic information (including self-rated disability status), disease characteristics, current MS-related and/or mood stabilizing medication and previous experience with psychological therapy, the Internet and mindfulness meditation (as described above). We will also be collecting self-report data measuring symptoms of depression, anxiety, fatigue, pain, quality of life and mindfulness. We will also collect information about the type of device (e.g. iPhone, iPad, android, computer) you are using to complete the online program, and how much time you spend on each webpage. **However, we will not be collecting information about your IP address or cookies.**

Your personal information will be kept confidential. The only times that we would need to break confidentiality would be:

1. In a situation where there is reason to believe that you or somebody else is in danger, in which case, supervision will be sought from Professor Louise Sharpe and we may need to talk to other people to ensure that nobody is harmed
2. In a situation where the study files are subpoenaed.

In rare cases such as these, we will notify you before any action is taken.

All electronic data will be stored securely and confidentially on the University of Sydney’s Classic Research Data Store, and will be retained for a minimum of 20 years. Only Amy-Lee Sesel and Professor Louise Sharpe (who are both psychologists and researchers on this study) will have access to your personal, identifying information, and this will be encrypted as an extra layer of security in our data management system.

De-identified data from this study will be published as part of Amy-Lee Sesel’s PhD project, as well as in peer-review journals, conferences, book chapters, and in an open access repository. We may also use this de-identified data in future projects (but will seek ethics approval before doing so).

By providing your consent, you are agreeing to us collecting personal information about you for the purposes of this research study. Your information will only be used for the purposes outlined in this Participant Information Statement, unless you consent otherwise.

1. **What will happen to my treatment when the study is finished?**

You will continue to have access to the online mindfulness program after the study finishes.

1. **Can I tell other people about the study?**

Yes, you are welcome to tell other people about the study.

1. **What if I would like further information about the study?**

When you have read this information, Amy-Lee Sesel will be available to discuss it with you further and answer any questions you may have. If you would like to know more at any stage during the study, please feel free to contact:

Amy-Lee Sesel (psychologist, and PhD researcher)

Email: [amy-lee.sesel@sydney.edu.au](mailto:amy-lee.sesel@sydney.edu.au)

Phone: +61417 06 1710

1. **Will I be told the results of the study?**

If you wish to receive feedback about the overall results of the study please indicate this below. The feedback will be sent to you when the study is finished, in the form of a one page lay summary of the study results. However please note, we will not be providing participants access to their individual scores on specific questionnaires.

1. **What if I have a complaint or any concerns about the study?**

Research involving humans in Australia is reviewed by an independent group of people called a Human Research Ethics Committee (HREC). The ethical aspects of this study have been approved by the HREC of the University of Sydney *[2018/402].* As part of this process, we have agreed to carry out the study according to the *National Statement on Ethical Conduct in Human Research (2007).* This statement has been developed to protect people who agree to take part in research studies.

If you are concerned about the way this study is being conducted or you wish to make a complaint to someone independent from the study, please contact the university using the details outlined below. Please quote the study title and protocol number.

The Manager, Ethics Administration, University of Sydney:

- - **Telephone:** +61 2 8627 8176
  - **Email:** [ro.humanethics@sydney.edu.au](mailto:ro.humanethics@sydney.edu.au)
  - **Fax:** +61 2 8627 8177 (Facsimile)

# This information sheet is for you to keep

| 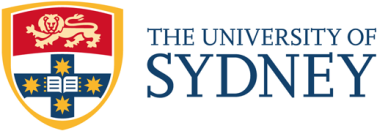 | |  | **School of Psychology Faculty of Science** |
| --- | --- | --- | --- |
|  | ABN 15 211 513 464 | |  |
|  | **Professor Louise Sharpe** | | Room 450  Brennan MacCallum (A18)  The University of Sydney  NSW 2006 AUSTRALIA  Telephone: +61 2 9351 4558  Email: louise.sharpe@sydney.edu.au  Web: <http://www.sydney.edu.au/> |

**Online Mindfulness Program for People with MS**

**ONLINE PARTICIPANT CONSENT FORM**

I agree to take part in this research study.

In giving my consent I state that:

- I understand the purpose of the study, what I will be asked to do, and any risks/benefits involved.
- I have read the Participant Information Statement and have been able to discuss my involvement in the study with the researchers if I wished to do so.
- I have contacted the researchers to answer any questions that I had about the study and I am happy with the answers provided.
- I understand that being in this study is completely voluntary and I do not have to take part. My decision whether to be in the study will not affect my relationship with the researchers or anyone else at the University of Sydney or the MS Clinic (Brain and Mind Centre) now or in the future.
- I understand that I can withdraw from the study at any time.
- I understand that my questionnaire responses cannot be withdrawn once they are submitted as they will become part of a pool of data and will be analysed accordingly.
- I understand that personal information about me that is collected over the course of this project will be stored securely and will only be used for purposes that I have agreed to. I understand that information about me will only be told to others with my permission, except as required by law.
- I understand that the results of this study may be published, used for future research (provided ethics approval is granted), submitted to a public research database and/or given to other researchers so that they can use the results in their research. However, these results will not contain my name or any identifiable information about me.
- I understand that if I do not consent to have my neurologist contacted, I will not be eligible to participate in the study. All information provided by my neurologist (with my permission) will be kept strictly confidential, and the researchers on this project with exclusive access to this information will be Professor Louise Sharpe (the Chief Investigator) and Amy-Lee Sesel (Masters of clinical psychology/ PhD candidate).
- Yes, I am happy for the researchers to contact my neurologist

Neurologist Name:___________________________________

Place of Work: ______________________________________

- No, I do not want the researchers to contact my neurologist and therefore I do not consent to participating in this clinical trial.

I consent to:

- **Being contacted about future studies** YES NO

//

**I would like to receive feedback about the overall results of this study** YES NO

If you clicked **YES**, please provide us with your e-mail address:

Email: ___________________________________________________

Please click on the relevant button:

/

/
